# Fertility in Ethiopia: factors associated with the number of live births —a survey—adjusted poisson regression analysis of the 2019 ethiopian mini demographic and health survey

**DOI:** 10.3389/frph.2026.1928107

**Published:** 2026-10-07

**Authors:** Melese Dereje Mesfin, Amare Yirga Wondu, Dahabo Adi Galgalo, Leman Atmaca, Ágnes Buda, Viktória Prémusz

**Affiliations:** 1 Doctoral School of Health Science, Faculty of Health Sciences, University of Pécs, Pecs, Hungary; 2 Department of Clinical Medical Sciences, Medical School, University of Pécs, Pécs, Hungary; 3 National Laboratory on Human Reproduction, University of Pécs, Pécs, Hungary; 4 Institute of Physiotherapy and Sport Sciences, Faculty of Health Sciences, University of Pécs, Pécs, Hungary; 5 Department of Obstetrics and Gynecology, Clinical Center, University of Pécs, Pécs, Hungary

**Keywords:** Ethiopia, Ethiopian mini demographic and health survey, fertility, live births, survey-adjusted poisson regression

## Abstract

**Background:**

Fertility is a key demographic and public health phenomenon reflecting actual childbearing within a population. It can be characterized using several measures, including total fertility rate, age-specific fertility rates, children ever born, and the number of births occurring within a defined reference period. These measures capture different dimensions of reproductive experience and are associated with a complex interplay of socioeconomic, demographic, cultural, biological, and policy-related factors.

**Methods:**

By dividing the nine regions into urban and rural categories, twenty-one sampling strata were created. The survey employed a two-stage stratified cluster sampling design. In the first stage, 305 enumeration areas (EAs) were selected from the national sampling frame using probability proportional to size. A total of 8,885 women were included in the descriptive analysis, while the primary multivariable analysis was restricted to 6,585 ever-married/partnered women. A survey-adjusted Poisson regression model was used to examine associations between explanatory variables and the number of live births during the five years preceding the survey. Statistical significance was assessed using a two-sided *p*-value of <0.05.

**Result and conclusion:**

The mean age of the participants was 27.6 years. More than four-fifths of women had either no formal education (40.4%) or primary education (41.7%). Compared with women aged 15–19 years, the expected number of recent live births was higher among women aged 20–24 years (aIRR = 2.129, 95% CI: 1.759–2.577). Women residing in Amhara had a lower expected number of recent live births than women residing in Tigray (aIRR = 0.765, 95% CI: 0.658–0.890). Lower expected counts were also observed in Addis Ababa (aIRR = 0.818, 95% CI: 0.684–0.979) and Dire Dawa (aIRR = 0.792, 95% CI: 0.659–0.952). An inverse association was observed between household wealth and recent live-birth counts. These findings highlight the multifactorial nature of recent live births in Ethiopia and suggest that fertility patterns are associated with a combination of individual, household, and contextual factors. These insights may help inform evidence-based reproductive health and family planning policies tailored to the Ethiopian context.

## 1 Introduction

Fertility is a central demographic and public health phenomenon referring to actual childbearing or reproductive performance within a population. It can be characterized using several demographic measures, including the total fertility rate (TFR), age-specific fertility rates, children ever born, and counts of births occurring within a specified reference period. These measures capture different dimensions and time frames of reproductive experience and should therefore be interpreted according to their specific definitions and reference periods. In demographic research, fertility as observed reproductive performance is conceptually distinct from fecundity, which refers to the biological capacity to reproduce. Fertility patterns are associated with a complex interplay of demographic, socioeconomic, cultural, biological, and reproductive health factors (1–4).

Total fertility rates vary substantially across countries and regions. Globally, the TFR has declined considerably over recent decades, although marked geographic differences remain. In many high-income countries, fertility has fallen below the replacement level of approximately 2.1 births per woman, whereas fertility remains comparatively higher in much of sub-Saharan Africa. Ethiopia has also experienced a substantial fertility decline, although important regional and socioeconomic differences persist (5–12).

In low- and lower-middle-income countries, high fertility and rapid population growth may present challenges to achieving the Sustainable Development Goals (SDGs), particularly those related to health, education, and gender equality (13).

Fertility patterns are associated with a broad range of socioeconomic, cultural, demographic, biological, health-related, and policy factors. The interaction of these characteristics contributes to substantial variation in childbearing patterns across populations (14). In many high-income settings, increased female labour-force participation, delayed marriage and childbearing, changing social norms, economic considerations, and access to family planning have been associated with declining fertility. Conversely, higher fertility levels remain common in many lower-income settings characterized by socioeconomic disadvantage, limited access to health services, and lower educational attainment. Sub-Saharan Africa continues to have some of the highest fertility levels globally, with countries such as Niger, Somalia, and Chad reporting particularly high fertility rates (15, 16).

Fertility in Ethiopia has declined substantially over recent decades, although considerable socioeconomic, demographic, and geographic variation remains (2, 17). Previous studies have examined fertility in Ethiopia using several demographic measures; however, relatively few have investigated factors associated specifically with the individual-level number of live births occurring within a recent, fixed 5-year reference period using nationally representative data while accounting for both the complex survey design and the count nature of the outcome. Distinguishing this measure from population-level fertility rates and lifetime measures such as children ever born is important because these indicators capture different dimensions and time frames of reproductive experience. Therefore, this study aimed to identify socioeconomic, demographic, and geographic factors associated with the number of live births during the five years preceding the 2019 EMDHS among reproductive-age women in Ethiopia.

## 2 Methods

Ethiopia is administratively divided into nine regional states and two city administrations. The 2019 Ethiopian Mini Demographic and Health Survey (EMDHS) was designed to generate nationally representative estimates of key demographic and health indicators for the country, with separate estimates for urban and rural populations and each of the nine regions and two city administrations. The survey employed a two-stage stratified cluster sampling design. In the first stage, 305 enumeration areas (EAs) were selected from the national sampling frame using probability proportional to size. In the second stage, a fixed number of households was selected systematically from each sampled EA.

This study was a secondary analysis of data obtained from the 2019 Ethiopian Mini Demographic and Health Survey (EMDHS). The analysis was conducted using the Individual Recode (IR) dataset, which contains one record for each interviewed woman of reproductive age (15–49 years). A total of 8,885 women were included in the descriptive analysis, of whom 6,585 ever-married/partnered women constituted the primary multivariable analytical sample. The EMDHS collected comprehensive information on fertility, reproductive health, socioeconomic characteristics, and other demographic indicators using the standard DHS Woman's Questionnaire (18).

### 2.1 Study variables

The outcome variable was the number of live births during the five years preceding the survey (EMDHS variable V208). This variable represents the individual-level count of live births reported by each interviewed woman during the fixed five-year reference period and should not be interpreted as a population-level fertility rate or lifetime fertility measure. Covariates for the multivariable model were selected based on their conceptual relevance to recent childbearing, their temporal relationship with the outcome, and evidence from previous literature (16–20). Current pregnancy status was retained as a descriptive reproductive characteristic but was not included in the primary multivariable model because it was measured at the time of the survey and had not necessarily resulted in a live birth during the five-year outcome period. In addition, current pregnancy may reflect recent reproductive history, making its temporal relationship with the outcome difficult to establish in this cross-sectional analysis. To assess the influence of this decision, a sensitivity analysis was conducted by additionally adjusting the primary model for current pregnancy status.

### 2.2 Method of data analysis

The 2019 Ethiopian Mini Demographic and Health Survey (EMDHS) employed a complex two-stage stratified cluster sampling design. The primary sampling unit (PSU) was specified using V021, the sampling strata using V022, and the individual sampling weight using V005. The sampling weight was rescaled by dividing V005 by 1,000,000 (wt = V005/1,000,000). The survey design was then declared in Stata using svyset v021 [pweight = wt], strata(v022). Survey-adjusted estimates and standard errors were calculated using Taylor-series linearization. The dataset included 305 PSUs distributed across 21 strata, with at least three PSUs in each stratum. Therefore, there were no singleton strata, and no additional adjustment for singleton strata was required. Descriptive statistics were used to summarize the characteristics of the study participants. The outcome was operationalized as the number of live births during the five years preceding the 2019 EMDHS (variable V208). Because the outcome variables represented count data. model adequacy and distributional assumptions were evaluated using complementray diagnostic and sensetivity analyses. Post-estimatio diagnostics indcated cionditional underdispersion in the primary analytical sample (Pearson dispersion statastic = 0.712; Deviamce/*df* = 0.795), rather than overdispersion. Anegative Binomial model was intially evaluated; however, its dispersion parameter approached the boundary at zero, and it didn't improve model fit relative to the conventional poisson model (AIC ∼ 14,210.71 vs. 14. 208.71).

Because the observed underdispersion could potentially affect the poisson distributional assumption, ad identically specified Generallized Pooisson model permitting underdisoerssion was additionally fitted as a likelihood-based sensitvity analysis. the generalized poisson dispersion parameter was negative (*α* = −0.182), consistent with the underdisperssion. Compared with the conventional Poisson Likelihood modelshowed improved improved distributionla fit (AIC = 13,641.11 vs. 14, 208.71; BIC = 13,892.44 vs. 14,453.24). Neverthelress, realxing the equi-disperssion assumption produced minimal changes in coefficent magnitudes, with absoulte relative changes in IRRs below 5% across all non-referende predictor categories (Supplementary Table S4).

A Zero Inflated Poisson model was also evaluated to assess whether excess zeros warranted a zero-inflated specification. The observed survey-adjusted proportion of zero count was lower than that predicted by the conventional Poisson model (40.46% vs. 46.89%). And the estimated structural-zero probability from the zero-inflated model was effectively zero (<0.01%). The zero-inflated specifications also did not improve AIC or BIC. Accordingly neither excess zero inflation nor overdisperssion was supported by data.

Comparsion of the observed Survey weighted and Posson model-predicted probabailities for live-birth counts from 0 to 5 showed somen dome differences across individual count categories (Supplementary Table S1). Therefore, the outcome was not assumed to follow a perfectly Poisson distribution. However, the survey-adjusted Poisson model was retained as the primary inferential model because it directly incorporated the EMDHS sampling weights, stratification, and clustering, with Taylor-series linearization for design-based variance estimationc (19, 20). The Generlized Poisson model was therefore used as a likelihood-based sensitivity analysis to assess whether the substantiative conclusions were robust to relaxation of equidisperssion assumption, rather than as a replacement for the primary design-based survey analysis.

Associations between the number of live births during the five years preceding the survey and the explanatory variables were estimated using survey-adjusted Poisson regression and are presented as adjusted incidence rate ratios (aIRRs) with corresponding 95% confidence intervals (CIs). Missing data were assessed for the outcome and all covariates included in the final model. Complete information was available for the outcome and covariates among all 8,885 women; therefore, no observations were excluded because of missing data. The primary multivariable analysis was restricted to 6,585 ever-married/partnered women to provide more interpretable estimates of the associations involving marital status, while the full sample of 8,885 reproductive-age women was retained for sensitivity analysis. The 15–19-year age group was selected as the reference category to provide a chronologically ordered comparison with the youngest reproductive-age group. Estimates for categories with relatively small numbers of observations were interpreted cautiously because of their potentially lower precision. Statistical significance was determined using a two-sided *p*-value of <0.05. All statistical analyses were performed using Stata version 17.0 (StataCorp LLC, College Station, TX, USA) (21). This study was reported in accordance with the Strengthening the Reporting of Observational Studies in Epidemiology (STROBE) Statement guidelines (22).

### 2.3 Operational definitions

#### 2.3.1 Household wealth index

A composite measure developed by the DHS Program using principal component analysis of household assets, housing characteristics, and access to utilities. Households were categorized into five wealth quintiles (poorest, poorer, middle, richer, and richest).

#### 2.3.2 Current contraceptive method use

Derived from DHS variable V313 (“current use by method type”) and categorized as no current method, modern method, or traditional/folkloric method. V364 (“contraceptive use and intention”) was not used in the revised multivariable analysis because, in this dataset, all women classified as using no method in V313 corresponded to the “does not intend to use” category in V364, preventing separate identification of non-users who intended future contraceptive use.

#### 2.3.3 Residence

Urban or rural according to the EMDHS classification.

#### 2.3.4 Recent live-birth count

The number of live births reported by an interviewed woman during the five years preceding the survey (EMDHS variable V208). In the present study, the term “recent live-birth count” is used as a shorthand for “the number of live births during the five years preceding the survey.”

### 2.4 Ethical considerations

This study used de-identified individual-level data from the 2019 Ethiopian Mini Demographic and Health Survey (EMDHS). The authors obtained authorization to access and use the dataset for secondary research. No participants were newly recruited or directly contacted, and no personally identifiable information was accessed. Ethical approval and informed consent for the original survey were obtained by the institutions responsible for conducting the EMDHS in accordance with the survey protocol.

## 3 Results

### 3.1 Socioeconomic, demographic, and reproductive characteristics of reproductive-age women in Ethiopia

A total of 8,885 women aged 15–49 years were included in the descriptive analysis. Complete information was available for the outcome and covariates included in the analysis; therefore, no observations were excluded because of missing data. The mean age of the participants was 27.6 years. Approximately two-thirds (67.8%) resided in rural areas. More than four-fifths of women had either no formal education (40.4%) or primary education (41.7%), whereas only 5.7% had attained higher education. Orthodox Christianity (41.5%) was the most frequently reported religion, followed by Islam (29.5%) and Protestant Christianity (27.4%). Oromia contributed the largest proportion of study participants (37.7%), followed by Amhara (22.8%) and the Southern Nations, Nationalities and Peoples' Region (19.2%). Nearly one-quarter (25.7%) of participants belonged to the richest wealth quintile, while 16.2% belonged to the poorest quintile. Most women were married (64.6%), 71.2% reported no current contraceptive method use, and 7.8% were pregnant at the time of the survey (Table 1).

**Table 1:** Socio-economic and demographic characteristics of reproductive age women EMDHS, 2019.

Characteristic	Category	Weighted *n*	Weighted %
Place of residence	Urban	2,861.2	32.2
	Rural	6,023.8	67.8
Educational attainment	No education	3,589.3	40.4
	Primary	3,700.8	41.7
	Secondary	1,088.2	12.2
	Higher	506.7	5.7
Religion	Orthodox	3,684.7	41.5
	Catholic	47.0	0.5
	Protestant	2,435.5	27.4
	Muslim	2,619.5	29.5
	Traditional	82.8	0.9
	Other	15.5	0.2
Wealth index	Poorest	1,436.7	16.2
	Poorer	1,615.3	18.2
	Middle	1,671.5	18.8
	Richer	1,874.3	21.1
	Richest	2,287.2	25.7
Marital status	Never in union	2,325.3	26.2
	Married	5,742.9	64.6
	Living with partner	121.1	1.4
	Widowed	185.3	2.1
	Divorced	377.1	4.2
	Separated	133.3	1.5
Current Contraceptive method use	Using modern method	2,494.7	28.1
	Using traditional method	61.0	0.7
	No current method	6,329.3	71.2
Current pregnancy	Yes	688.9	7.8
	No/Unsure	8,196.1	92.2
Region	Tigray	628.9	7.1
	Afar	85.5	1.0
	Amhara	2,025.8	22.8
	Oromia	3,347.0	37.7
	Somali	420.3	4.7
	Benishangul-Gumuz	98.4	1.1
	SNNPR	1,705.3	19.2
	Gambela	40.5	0.5
	Harari	26.9	0.3
	Addis Ababa	442.0	5.0
	Dire Dawa	64.5	0.7

SNNPR, Southern Nations Nationalities and People Region.

### 3.2 Number of live births during the five years preceding the survey

The number of live births during the five years preceding the survey ranged from 0 to 5. More than half of the women (55.8%) reported no live births during the reference period, whereas 28.2% and 14.1% reported one and two live births, respectively. Only 1.9% of participants reported three or more live births during the five-year period. The mean number of live births was 0.62 (median = 0, variance = 0.64), indicating that most women had no or only one recent live birth (Table 2).

**Table 2:** Distribution of the number of live births during the five years preceding the survey among reproductive-age women in Ethiopia, 2019 EMDHS.

Number of live births	Weighted n	Weighted %
0	4,958.4	55.8
1	2,505.8	28.2
2	1,254.5	14.1
3	156.4	1.8
4	6.5	0.1
5	3.4	<0.1

### 3.3 Factors associated with the number of live births during the five years preceding the survey among reproductive-age women in Ethiopia

Among the 2,300 women classified as never in union, only 29 (1.26%) reported at least one live birth during the five years preceding the survey. Consequently, comparisons using never-in-union women as the reference group produced very large incidence-rate ratios. The primary multivariable analysis was therefore restricted to the 6,585 women who had ever been married or partnered. This restricted sample retained all 305 PSUs and 21 sampling strata. Survey-adjusted multivariable Poisson regression was used to examine factors associated with the number of live births during the five years preceding the survey. Age group, region, household wealth, religion, marital status, and current contraceptive method use were jointly associated with the outcome, whereas place of residence and educational attainment were not independently associated after adjustment for the other covariates. The design-adjusted omnibus tests were statistically significant for age group (Wald *χ*^2^ = 263.34, *p* < 0.001), region (Wald *χ*^2^ = 26.92, *p* = 0.003), wealth index (Wald *χ*^2^ = 33.12, *p* < 0.001), religion (Wald *χ*^2^ = 29.47, *p* < 0.001), marital status (Wald *χ*^2^ = 243.67, *p* < 0.001), and current contraceptive method use [*F*(2, 284) = 3.22, *p* = 0.041].

Compared with women aged 15–19 years, the expected number of recent live births was higher among women aged 20–24 years (aIRR = 2.129, 95% CI: 1.759–2.577), 25–29 years (aIRR = 2.447, 95% CI: 2.010–2.978), 30–34 years (aIRR = 2.169, 95% CI: 1.780–2.643), and 35–39 years (aIRR = 1.611, 95% CI: 1.310–1.980). Women aged 45–49 years had a lower expected birth count than those aged 15–19 years (aIRR = 0.434, 95% CI: 0.315–0.597), whereas the difference for women aged 40–44 years was not statistically significant.

Compared with women residing in Tigray, women in Amhara had a 24.0% lower expected number of recent live births (aIRR = 0.765, 95% CI: 0.658–0.890). Lower expected counts were also observed in Addis Ababa (aIRR = 0.818, 95% CI: 0.684–0.979) and Dire Dawa (aIRR = 0.792, 95% CI: 0.659–0.952). The remaining regional comparisons were not statistically significant. Rural residence was not associated with the outcome after adjustment (aIRR = 1.002, 95% CI: 0.867–1.158).

An inverse association was observed between household wealth and the number of live births during the five years preceding the survey. Compared with women in the poorest wealth quintile, women in the middle, richer, and richest quintiles had 15% (aIRR = 0.853, 95% CI: 0.751–0.968), 25% (aIRR = 0.756, 95% CI: 0.662–0.863), and 32.5% (aIRR = 0.675, 95% CI: 0.579–0.786) lower expected birth counts, respectively. The difference between the poorer and poorest quintiles was not statistically significant whereas educational attainment was not independently associated with the outcome. Compared with Orthodox Christian women, Catholic women had a lower expected number of recent live births (aIRR = 0.622, 95% CI: 0.443–0.872), whereas Muslim women (aIRR = 1.247, 95% CI: 1.063–1.465) and women reporting a traditional religion (aIRR = 1.451, 95% CI: 1.156–1.822) had higher expected counts.

Current modern contraceptive method use showed a small positive association with recent live-birth counts in the primary model. However, this association was attenuated and was no longer statistically significant when current pregnancy status was included in a sensitivity analysis. This lack of robustness, together with the different reference periods of the exposure and outcome, indicates that the finding should be interpreted cautiously. Current contraceptive method use and pregnancy status were measured at the time of the survey, whereas the outcome represents births occurring during the preceding five years. Therefore, temporal ordering cannot be established, and the observed relationship may reflect recent reproductive history, subsequent contraceptive adoption, or other unmeasured factors rather than an effect of contraception on childbearing.

Among ever-married/partnered women, marital status was associated with the expected number of recent live births. Compared with currently married women, women living with a partner had a lower expected birth count (aIRR = 0.378, 95% CI: 0.215–0.665; *p* < 0.001), as did widowed women (aIRR = 0.531, 95% CI: 0.364–0.776; *p* = 0.001) and divorced women (aIRR = 0.538, 95% CI: 0.434–0.666; *p* < 0.001). The association for separated women was not statistically significant (aIRR = 0.702, 95% CI: 0.464–1.064; *p* = 0.095) (Table 3).

**Table 3:** Factors associated with the number of live births during the five years preceding the survey among ever-married/partnered women in Ethiopia: survey-adjusted poisson regression, 2019 EMDHS.

Predictor	Category	aIRR	95% CI	*p*-value
Age group	15–19	Reference	—	—
	20–24	2.129	1.759–2.577	<0.001
	25–29	2.447	2.010–2.978	<0.001
	30–34	2.169	1.780–2.643	<0.001
	35–39	1.611	1.310–1.980	<0.001
	40–44	1.058	0.811–1.379	0.678
	45–49	0.434	0.315–0.597	<0.001
Residence	Urban	Reference	—	—
	Rural	1.002	0.867–1.158	0.978
Region	Tigray	Reference	—	—
	Afar	0.956	0.779–1.173	0.665
	Amhara	0.765	0.658–0.890	<0.001
	Oromia	0.887	0.725–1.085	0.241
	Somali	1.038	0.845–1.276	0.720
	Benishangul-Gumuz	0.882	0.753–1.033	0.120
	SNNPR	0.902	0.746–1.090	0.284
	Gambela	0.867	0.700–1.074	0.190
	Harari	0.914	0.762–1.096	0.328
	Addis Ababa	0.818	0.684–0.979	0.029
	Dire Dawa	0.792	0.659–0.952	0.013
Education	No education	Reference	–	-
	Primary	0.938	0.861–1.021	0.137
	Secondary	0.927	0.823–1.044	0.208
	Higher	0.888	0.725–1.087	0.247
Wealth index	Poorest	Reference	–	–
	Poorer	0.950	0.854–1.058	0.351
	Middle	0.853	0.751–0.968	0.014
	Richer	0.756	0.662–0.863	<0.001
	Richest	0.675	0.579–0.786	<0.001
Religion	Orthodox	Reference	–	–
	Catholic	0.622	0.443–0.872	0.006
	Protestant	1.054	0.897–1.240	0.520
	Muslim	1.247	1.063–1.465	0.007
	Traditional	1.451	1.156–1.822	0.001
	Other	1.009	0.745–1.367	0.955
Marital status	Married	Reference	—	—
	Living with partner	0.378	0.215–0.665	<0.001
	Widowed	0.531	0.364–0.776	0.001
	Divorced	0.538	0.434–0.666	<0.001
	Separated/no longer living together	0.702	0.464–1.064	0.095
Current contraceptive method use	No current method	Reference	—	—
	Modern method	1.069	1.011–1.131	0.019
	Traditional/folkloric method	0.820	0.480–1.402	0.468

Primary analysis restricted to women who had ever been married or partnered (*n* = 6,585). Survey-adjusted estimates accounted for sampling weights, 305 PSUs, and 21 strata; design degrees of freedom = 284. Current contraceptive method use was derived from V313. Traditional and folkloric methods were combined because only one respondent reported folkloric-method use.

aIRR, adjusted incidence rate ratio; CI, confidence interval.

The primary multivariable analysis was restricted to women who had ever been married or partnered because recent childbearing was extremely uncommon among women who had never entered a union. Among the 2,300 women classified as never in union, only 29 (1.26%) reported at least one live birth during the five years preceding the survey. In a sensitivity analysis additionally adjusting the primary model for current pregnancy status, estimates for the principal sociodemographic factors remained largely unchanged (Supplementary Table S3). Current pregnancy was associated with a lower expected number of live births during the five years preceding the survey (aIRR = 0.872, 95% CI: 0.783–0.972; *p* = 0.013). The association between current modern contraceptive method use and recent live-birth counts observed in the primary model (aIRR = 1.069; *p* = 0.019) was attenuated and was no longer statistically significant after adjustment for current pregnancy status (aIRR = 1.036; *p* = 0.230). In the full-sample sensitivity analysis (*n* = 8,885), the direction and magnitude of the associations for the principal non-marital covariates were generally consistent with those of the primary analysis (Supplementary Table S2).

## 4 Discussion

This study examined socioeconomic, demographic, and geographic factors associated with the number of live births during the five years preceding the 2019 Ethiopian Mini Demographic and Health Survey. Using survey-adjusted Poisson regression, the analysis accounted for the count nature of the outcome and the complex sampling design. Maternal age, region, household wealth, religion, marital status, and current contraceptive method use were independently associated with recent live-birth counts, whereas place of residence and educational attainment were not independently associated after adjustment for the other characteristics included in the model.

The findings demonstrate considerable variation in child-bearing across socioeconomic, demographic, cultural, and geographic groups in Ethiopia. More than half of the women had no live births during the five years preceding the survey, while approximately 28% had one birth and 14% had two births. The mean number of recent live births was relatively low, which should be interpreted in the context of the limited five-year reference period and the broader decline in fertility documented in Ethiopia. This estimate should not be directly compared with studies that used children ever born or completed family size, because those measures capture cumulative reproductive experience across a woman's reproductive life. By focusing on the total number of live births during the 5 years preceding the survey, the present study provides a more contemporary measure of fertility patterns and their associations with current socioeconomic and demographic conditions.

Previous studies using the 2019 EMDHS have examined factors assocaited with cildbearing primarility using the number of chlidrennever biorn as thre outcome (2, 23, 24). Severla findings were consistent with the present analysis. For example, Muslim women had higher recent live birth counts than Orthodox women in our study(aIRR = 1.247, 95% CI: 1.063–1.465), consistent with the postive asocciations reported by Taye et wla and Yirsaw et al. (IRR = 1.13, 95% CI: 1.08–1.18) abd Gebre et al. (aIRR = −1.096, 95% CI: 1.043–1.151). Household wealth also showed a consistently inverse association with childbearing, although the magnitude and categorization of wealth differed across studies. In contrast, educational attinment was nott independently assocaited with recent live births counts in our analysis (higher Vs no education. aIRR = 0.88. 95% CI: 0.725–1.087), whereas previious analysis of of children ever born reported substantially lower counts among more highly educated women. Place of residence similarly shoed no independent association in our study (rural vs. urban: Airr = 1.002, 95% CI: 0.867–1.158), whlemTaye et al. Yirsaw eta al reported higher cumilative childbearing among rural women (IRSS = −1.09, 95% CI: 1.05–1.14); noatably, Gebret et al. also found no significant adjusted rural-urban difference. The simiilarities and differences may reflect variation in the nalytical poulation and model specification and, importantly, the outcome timegrame: children ever born captiuress cumilative reproductive experience, whereas the present study focuses on births occuring within a fixed five-year period.

Childbearing patterns are associated with a complex combination of biological, reproductive, socioeconomic, cultural, partnership, and health-service characteristics. Factors such as age, timing of marriage and first birth, household resources, contraceptive behavior, child mortality, education, and geographic context may be associated with differences in both childbearing opportunities and reproductive decisions. The associations identified in the present study should therefore be interpreted as interrelated population-level patterns rather than isolated or necessarily causal effects (14).

Maternal age showed one of the strongest associations with recent live-birth counts. Compared with adolescents aged 15–19 years, women aged 20–39 years had significantly higher expected recent live-birth counts during the five years preceding the survey, with the highest fertility observed among women aged 25–29 years. The expected number of recent live births subsequently declined at older ages; women aged 40–44 years did not differ significantly from the 15–19-year reference group, while women aged 45–49 years had a lower expected recent live-birth count. This age pattern is consistent with the natural reproductive life course, whereby fertility typically increases following marriage and family formation, reaches its peak during the late twenties and early thirties, and gradually declines with advancing age because of reduced fecundity, completion of desired family size, and increased adoption of fertility-limiting behaviours. Similar age-specific fertility patterns have been reported in Ethiopia and other low- and middle-income countries, highlighting maternal age as one of the most consistent demographic predictors of fertility (2, 7, 18, 23, 25–27). These findings emphasize the importance of ensuring that reproductive health and family planning services are responsive to women's changing reproductive needs throughout the life course.

Contrary to findings from several previous studies conducted in Ethiopia and other low- and middle-income countries, place of residence was not independently associated with the number of live births during the five years preceding the survey after adjustment for socioeconomic, demographic, and geographic characteristics. While crude comparisons frequently demonstrate higher fertility among rural women, the lack of an independent association after multivariable adjustment suggests that crude urban–rural differences may partly reflect differences in household wealth, regional characteristics, marital patterns, and other population characteristics. This finding highlights the importance of accounting for socioeconomic and contextual characteristics when examining urban–rural differences in reproductive outcomes. Similar attenuation of the urban-rural association following multivariable adjustment has been reported in other nationally representative demographic studies (2, 14, 17, 18).

Household wealth was inversely associated with recent live-birth counts. The inverse association with household wealth may reflect differences in educational and employment opportunities, access to reproductive health services and modern contraceptive methods, and reproductive decision-making autonomy, as suggested by demographic transition theory and previous research. Our findings are consistent with previous studies conducted in Ethiopia and other low- and middle-income countries that reported declining fertility with increasing household socioeconomic status (2, 10, 14, 17).

Significant regional differences in recent live-birth counts were observed after adjustment for individual socioeconomic and demographic characteristics. Women residing in Amhara, Addis Ababa, and Dire Dawa had lower expected recent live-birth counts than women in the reference region, Tigray. These differences may be associated with contextual factors such as access to reproductive health services, urbanization, educational and employment opportunities, and reproductive preferences, as suggested by previous studies. However, these mechanisms were not directly measured in the present analysis and therefore cannot be established from our findings (2, 8, 14, 17, 18).

Differences in recent live-birth counts were also observed across religious groups. Compared with Orthodox women, Muslim women had a higher expected recent birth count. Previous research has suggested that reproductive preferences, marriage patterns, contraceptive practices, and sociocultural norms may vary across religious and social contexts and could potentially contribute to such differences. However, these mechanisms were not directly measured in the present study; therefore, religion should be interpreted as a broad sociodemographic marker rather than evidence of a specific causal pathway. Because some religious groups constituted relatively small proportions of the study population, these findings should be interpreted with appropriate caution (2, 14, 17, 18, 28).

Marital status was associated with recent live-birth counts among ever-married or partnered women. Compared with currently married women, those living with a partner, widowed, or divorced had lower expected numbers of live births during the five years preceding the survey. These findings should be interpreted primarily in relation to differences in union continuity and exposure to childbearing opportunity rather than as causal effects of marital status itself. The primary analysis was restricted to ever-married/partnered women because recent childbearing was extremely uncommon among women who had never entered a union. In the full sample, using never-in-union women as the reference category consequently produced very large incidence-rate ratios. The sensitivity analysis nevertheless showed that excluding this near-structural reference group did not materially alter the direction or magnitude of the major associations observed for the other covariates (2, 8, 14, 17, 18).

Current modern contraceptive method use was associated with a slightly higher expected number of live births during the preceding five years compared with current non-use. This association should not be interpreted as indicating that contraceptive use increases fertility. Contraceptive use was measured at the time of the survey, whereas the outcome represents births accumulated over the preceding five years. Women who had recently achieved their desired births may have subsequently initiated contraception, creating temporal ambiguity and the possibility of reverse causation. The observed association should therefore be interpreted as a cross-sectional relationship between current contraceptive status and recent childbearing history rather than evidence of an effect of contraception on fertility (2, 18, 29–31).

## 5 Strength and limitations

### 5.1 Strengths

The present study has several important strengths. It utilized nationally representative data from the 2019 Ethiopian Mini Demographic and Health Survey and appropriately accounted for the complex survey design through the application of sampling weights, stratification, and clustering. The use of survey-adjusted Poisson regression, supported by model-diagnostic and model-comparison procedures, provided an appropriate analytical framework for examining the count outcome. In addition, sensitivity analyses using the full sample of reproductive-age women and additional adjustment for current pregnancy status allowed assessment of the robustness of the principal findings. The primary analysis retained all 305 PSUs and 21 sampling strata, thereby preserving the survey-design structure within the restricted ever-married/partnered analytical sample.

### 5.2 Limitations

Despite these strengths, several limitations should be acknowledged. First, the cross-sectional design precludes causal inference and does not permit determination of the temporal sequence between the explanatory variables and recent live-birth counts. This limitation is particularly relevant to current contraceptive method use and current pregnancy status, which were measured at the time of the survey, whereas the outcome captured births occurring during the preceding five years. Second, some variables were self-reported and may therefore be subject to recall or social-desirability bias. Third, the EMDHS does not capture several factors that may be associated with recent childbearing, including fertility intentions, infertility, partner characteristics, duration of marriage or union, and detailed measures of access to reproductive health services; consequently, residual confounding cannot be excluded. Fourth, the primary multivariable analysis was restricted to ever-married/partnered women because recent childbearing was extremely uncommon among never-in-union women. Therefore, the primary-model estimates are most directly applicable to ever-married/partnered reproductive-age women, although the full-sample sensitivity analysis produced broadly consistent findings for the principal non-marital covariates. The association between current contraceptive method use and recent live-birth counts was also sensitive to adjustment for current pregnancy status, further limiting causal or temporal interpretation of this finding. Finally, although the study examined the number of live births during the preceding five years to provide a measure of recent childbearing, this outcome is not directly comparable with lifetime measures such as children ever born or population-level indicators such as the total fertility rate.

## 6 Conclusion

This study identified several socioeconomic, demographic, and geographic factors associated with the number of live births during the five years preceding the 2019 Ethiopian Mini Demographic and Health Survey. Maternal age, household wealth, region, religion, and marital status were associated with recent live-birth counts, whereas educational attainment and place of residence were not independently associated after adjustment for the other characteristics included in the model. The observed association with current contraceptive method use should be interpreted cautiously because it was sensitive to adjustment for current pregnancy status. These findings underscore the multifactorial nature of recent childbearing and demonstrate that differences in recent live-birth counts are associated with a combination of individual, household, and contextual characteristics.

The observed regional and socioeconomic differences suggest that reproductive health and family planning strategies may benefit from attention to local demographic, cultural, and socioeconomic contexts. Strengthening equitable access to comprehensive reproductive health services, promoting informed reproductive decision-making, and addressing broader social determinants of reproductive health remain important public health priorities. Future longitudinal studies incorporating fertility intentions, partnership characteristics, and access to reproductive health services are warranted to better understand the temporal and causal pathways associated with recent childbearing and to inform evidence-based reproductive health policies.

## Data Availability

Publicly available datasets were analyzed in this study. This data can be found here: https://dhsprogram.com.
