# Supplementary material for: Fertility in Ethiopia: factors associated with the number of live births —a survey—adjusted poisson regression analysis of the 2019 ethiopian mini demographic and health survey
Source: Front Reprod Health. 2026 Oct 7;8:1928107. doi: 10.3389/frph.2026.1928107 (PMC13683705; doi:10.3389/frph.2026.1928107)
Supplement: Supplementary file 1 [file Table1.docx]

Supplemntary table S1 : Observed and Poisson Model-Predicted Distribution of Live Births During the Five Years Preceding the Survey Among Ever-Married/Partnered Women, 2019 EMDHS

| **Live births** | **Observed weighted %** | **Poisson-predicted %** |
| --- | --- | --- |
| 0 | 40.46 | 46.89 |
| 1 | 37.91 | 31.88 |
| 2 | 19.10 | 14.25 |
| 3 | 2.38 | 5.01 |
| 4 | 0.10 | 1.48 |
| 5 | 0.05 | 0.38 |
