# Supplementary material for: Fertility in Ethiopia: factors associated with the number of live births —a survey—adjusted poisson regression analysis of the 2019 ethiopian mini demographic and health survey
Source: Front Reprod Health. 2026 Oct 7;8:1928107. doi: 10.3389/frph.2026.1928107 (PMC13683705; doi:10.3389/frph.2026.1928107)
Supplement: Supplementary file 2 [file Table2.docx]

Supplementary Table S2. Sensitivity Analysis Comparing the Primary Ever-Married/Partnered Model with the Full-Sample Model Among Reproductive-Age Women, 2019 EMDHS

| **Predictor** | **Category** | **Primary aIRR** | **Primary 95% CI** | **p** | **Sensitivity aIRR** | **Sensitivity 95% CI** | **p** |
| --- | --- | --- | --- | --- | --- | --- | --- |
| Age group | 15–19 | Reference | -- | -- | Reference | -- | -- |
|  | 20–24 | 2.129 | 1.759–2.577 | <0.001 | 2.168 | 1.795–2.620 | <0.001 |
|  | 25–29 | 2.447 | 2.010–2.978 | <0.001 | 2.508 | 2.066–3.045 | <0.001 |
|  | 30–34 | 2.169 | 1.780–2.643 | <0.001 | 2.236 | 1.840–2.717 | <0.001 |
|  | 35–39 | 1.611 | 1.310–1.980 | <0.001 | 1.650 | 1.342–2.029 | <0.001 |
|  | 40–44 | 1.058 | 0.811–1.379 | 0.678 | 1.088 | 0.838–1.413 | 0.523 |
|  | 45–49 | 0.434 | 0.315–0.597 | <0.001 | 0.445 | 0.324–0.610 | <0.001 |
| Residence | Urban | Reference | -- | -- | Reference | -- | -- |
|  | Rural | 1.002 | 0.867–1.158 | 0.978 | 1.009 | 0.873–1.166 | 0.904 |
| Region | Tigray | Reference | -- | -- | Reference | -- | -- |
|  | Afar | 0.956 | 0.779–1.173 | 0.665 | 0.955 | 0.778–1.172 | 0.658 |
|  | Amhara | 0.765 | 0.658–0.890 | <0.001 | 0.760 | 0.654–0.884 | <0.001 |
|  | Oromia | 0.887 | 0.725–1.085 | 0.241 | 0.882 | 0.721–1.079 | 0.221 |
|  | Somali | 1.038 | 0.845–1.276 | 0.720 | 1.036 | 0.844–1.273 | 0.733 |
|  | Benishangul-Gumuz | 0.882 | 0.753–1.033 | 0.120 | 0.883 | 0.753–1.035 | 0.124 |
|  | SNNPR | 0.902 | 0.746–1.090 | 0.284 | 0.898 | 0.743–1.084 | 0.262 |
|  | Gambela | 0.867 | 0.700–1.074 | 0.190 | 0.885 | 0.716–1.093 | 0.254 |
|  | Harari | 0.914 | 0.762–1.096 | 0.328 | 0.907 | 0.756–1.087 | 0.290 |
|  | Addis Ababa | 0.818 | 0.684–0.979 | 0.029 | 0.815 | 0.682–0.975 | 0.025 |
|  | Dire Dawa | 0.792 | 0.659–0.952 | 0.013 | 0.794 | 0.660–0.954 | 0.014 |
| Education | No education | Reference | -- | -- | Reference | -- | -- |
|  | Primary | 0.938 | 0.861–1.021 | 0.137 | 0.941 | 0.864–1.024 | 0.159 |
|  | Secondary | 0.927 | 0.823–1.044 | 0.208 | 0.924 | 0.820–1.040 | 0.188 |
|  | Higher | 0.888 | 0.725–1.087 | 0.247 | 0.889 | 0.729–1.085 | 0.247 |
| Wealth index | Poorest | Reference | -- | -- | Reference | -- | -- |
|  | Poorer | 0.950 | 0.854–1.058 | 0.351 | 0.953 | 0.856–1.060 | 0.374 |
|  | Middle | 0.853 | 0.751–0.968 | 0.014 | 0.853 | 0.752–0.969 | 0.015 |
|  | Richer | 0.756 | 0.662–0.863 | <0.001 | 0.756 | 0.662–0.863 | <0.001 |
|  | Richest | 0.675 | 0.579–0.786 | <0.001 | 0.678 | 0.583–0.790 | <0.001 |
| Religion | Orthodox | Reference | -- | - | Reference | -- | -- |
|  | Catholic | 0.622 | 0.443–0.872 | 0.006 | 0.618 | 0.442–0.864 | 0.005 |
|  | Protestant | 1.054 | 0.897–1.240 | 0.520 | 1.055 | 0.898–1.239 | 0.515 |
|  | Muslim | 1.247 | 1.063–1.465 | 0.007 | 1.242 | 1.058–1.458 | 0.008 |
|  | Traditional | 1.451 | 1.156–1.822 | 0.001 | 1.444 | 1.150–1.814 | 0.002 |
|  | Other | 1.009 | 0.745–1.367 | 0.955 | 1.008 | 0.746–1.363 | 0.959 |
| Marital status | Never in union | Not included | -- | -- | Reference | -- | -- |
|  | Married | Reference | -- | -- | 60.835 | 34.093–108.554 | <0.001 |
|  | Living with partner | 0.378 | 0.215–0.665 | <0.001 | 23.064 | 10.046–52.953 | <0.001 |
|  | Widowed | 0.531 | 0.364–0.776 | 0.001 | 32.335 | 17.129–61.040 | <0.001 |
|  | Divorced | 0.538 | 0.434–0.666 | <0.001 | 32.786 | 17.758–60.530 | <0.001 |
|  | Separated/no longer living together | 0.702 | 0.464–1.064 | 0.095 | 42.848 | 21.129–86.894 | <0.001 |
| Current contraceptive method use | No method | Reference | -- | -- | Reference | -- | -- |
|  | Modern method | 1.069 | 1.011–1.131 | 0.019 | 1.073 | 1.014–1.136 | 0.016 |
|  | Traditional/folkloric method | 0.820 | 0.480–1.402 | 0.468 | 0.820 | 0.481–1.398 | 0.464 |

footnote: Primary analysis: ever-married/ever-partnered women, n = 6,585; 305 PSUs; 21 strata; design df = 284. Sensitivity analysis: all reproductive-age women, n = 8,885; 305 PSUs; 21 strata; design df = 284. Marital-status estimates are not directly comparable across models because the reference group differs: currently married women are the reference in the primary model, whereas never-in-union women are the reference in the full-sample sensitivity model.
