# Supplementary material for: Fertility in Ethiopia: factors associated with the number of live births —a survey—adjusted poisson regression analysis of the 2019 ethiopian mini demographic and health survey
Source: Front Reprod Health. 2026 Oct 7;8:1928107. doi: 10.3389/frph.2026.1928107 (PMC13683705; doi:10.3389/frph.2026.1928107)
Supplement: Supplementary file 3 [file Table3.docx]

Supplementary Table S3: Sensitivity Analysis of the Primary Survey-Adjusted Poisson Regression Model Additionally Adjusted for Current Pregnancy Status Among Ever-Married/Partnered Women, 2019 EMDHS

| **Variable** | **Category** | **Primary model aIRR (95% CI)** | **Pregnancy-adjusted aIRR (95% CI)** | **p-value*** |
| --- | --- | --- | --- | --- |
| **Age group** | 15–19 | Reference | Reference | -- |
|  | 20–24 | 2.129 (1.759–2.577) | **2.136 (1.764–2.587)** | <0.001 |
|  | 25–29 | 2.447 (2.012–2.976) | **2.456 (2.015–2.994)** | <0.001 |
|  | 30–34 | 2.169 (1.782–2.641) | **2.164 (1.776–2.638)** | <0.001 |
|  | 35–39 | 1.611 (1.311–1.979) | **1.599 (1.299–1.968)** | <0.001 |
|  | 40–44 | 1.058 (0.812–1.378) | **1.043 (0.800–1.359)** | 0.757 |
|  | 45–49 | 0.434 (0.315–0.596) | **0.425 (0.309–0.585)** | <0.001 |
| **Residence** | Urban | Reference | Reference | -- |
|  | Rural | 1.002 (0.868–1.157) | **1.003 (0.869–1.157)** | 0.968 |
| **Region** | Tigray | Reference | Reference | -- |
|  | Afar | 0.956 (0.780–1.172) | **0.948 (0.774–1.162)** | 0.609 |
|  | Amhara | 0.765 (0.658–0.889) | **0.764 (0.657–0.887)** | <0.001 |
|  | Oromia | 0.887 (0.725–1.084) | **0.885 (0.724–1.081)** | 0.231 |
|  | Somali | 1.038 (0.846–1.275) | **1.035 (0.843–1.270)** | 0.742 |
|  | Benishangul-Gumuz | 0.882 (0.753–1.033) | **0.878 (0.751–1.028)** | 0.105 |
|  | SNNPR | 0.902 (0.746–1.089) | **0.899 (0.745–1.086)** | 0.271 |
|  | Gambela | 0.867 (0.700–1.073) | **0.858 (0.695–1.059)** | 0.155 |
|  | Harari | 0.914 (0.762–1.095) | **0.916 (0.764–1.097)** | 0.340 |
|  | Addis Ababa | 0.818 (0.684–0.979) | **0.818 (0.684–0.979)** | 0.028 |
|  | Dire Dawa | 0.792 (0.659–0.951) | **0.790 (0.658–0.948)** | 0.011 |
| **Education** | No education | Reference | Reference | -- |
|  | Primary | 0.938 (0.861–1.020) | **0.941 (0.864–1.024)** | 0.160 |
|  | Secondary | 0.927 (0.823–1.043) | **0.930 (0.827–1.046)** | 0.226 |
|  | Higher | 0.888 (0.726–1.086) | **0.897 (0.734–1.098)** | 0.292 |
| **Household wealth** | Poorest | Reference | Reference | -- |
|  | Poorer | 0.950 (0.854–1.057) | **0.946 (0.850–1.054)** | 0.317 |
|  | Middle | 0.853 (0.752–0.968) | **0.852 (0.751–0.967)** | 0.013 |
|  | Richer | 0.756 (0.662–0.863) | **0.753 (0.659–0.860)** | <0.001 |
|  | Richest | 0.675 (0.579–0.786) | **0.675 (0.580–0.785)** | <0.001 |
| **Religion** | Orthodox | Reference | Reference | -- |
|  | Catholic | 0.622 (0.444–0.871) | **0.622 (0.444–0.872)** | 0.006 |
|  | Protestant | 1.054 (0.897–1.239) | **1.059 (0.900–1.246)** | 0.490 |
|  | Muslim | 1.247 (1.063–1.464) | **1.249 (1.064–1.465)** | 0.006 |
|  | Traditional | 1.451 (1.157–1.820) | **1.484 (1.167–1.887)** | 0.001 |
|  | Other | 1.009 (0.746–1.365) | **1.023 (0.753–1.389)** | 0.885 |
| **Marital status** | Married | Reference | Reference | -- |
|  | Living with partner | 0.378 (0.216–0.663) | **0.372 (0.212–0.653)** | <0.001 |
|  | Widowed | 0.531 (0.364–0.775) | **0.519 (0.353–0.761)** | <0.001 |
|  | Divorced | 0.538 (0.435–0.665) | **0.526 (0.424–0.653)** | <0.001 |
|  | Separated | 0.702 (0.464–1.062) | **0.686 (0.454–1.036)** | 0.073 |
| **Current contraceptive method** | No method | Reference | Reference | — |
|  | Traditional/folkloric | 0.820 (0.481–1.399) | **0.791 (0.461–1.356)** | 0.394 |
|  | Modern method | 1.069 (1.011–1.131) | **1.036 (0.978–1.098)** | 0.230 |
| **Current pregnancy** | No/unsure | -- | Reference | — |
|  | Currently pregnant | -- | **0.872 (0.783–0.972)** | **0.013** |
