# Supplementary material for: Fertility in Ethiopia: factors associated with the number of live births —a survey—adjusted poisson regression analysis of the 2019 ethiopian mini demographic and health survey
Source: Front Reprod Health. 2026 Oct 7;8:1928107. doi: 10.3389/frph.2026.1928107 (PMC13683705; doi:10.3389/frph.2026.1928107)
Supplement: Supplementary file 4 [file Table4.docx]

Supplementary Table S4: Sensitivity analysis comparing survey-adjusted Poisson, conventional Poisson, and generalized Poisson regression estimates among ever-married/partnered women, 2019 EMDHS (n = 6,585)

**Panel A**

| **Predictor** | **Category** | **Survey-adjusted Poisson aIRR (95% CI)** | **Conventional Poisson IRR** | **Generalized Poisson IRR** | **Relative change, %** |
| --- | --- | --- | --- | --- | --- |
| Age group | 15–19 | Reference | Reference | Reference | — |
|  | 20–24 | 2.129 (1.759–2.577) | 2.017 | 2.040 | +1.13 |
|  | 25–29 | 2.447 (2.010–2.978) | 2.262 | 2.283 | +0.91 |
|  | 30–34 | 2.169 (1.780–2.643) | 2.090 | 2.115 | +1.19 |
|  | 35–39 | 1.611 (1.310–1.980) | 1.492 | 1.515 | +1.55 |
|  | 40–44 | 1.058 (0.811–1.379) | 0.980 | 0.999 | +2.01 |
|  | 45–49 | 0.434 (0.315–0.597) | 0.452 | 0.462 | +2.18 |
| Residence | Urban | Ref. | Ref. | Ref. | -- |
|  | Rural | 1.002 (0.867–1.158) | 1.042 | 1.053 | +1.02 |
| Region | Tigray | Ref. | Ref. | Ref. | -- |
|  | Afar | 0.956 (0.779–1.173) | 0.852 | 0.880 | +3.25 |
|  | Amhara | 0.765 | 0.782 | 0.779 | −0.28 |
|  | Oromia | 0.887 | 0.836 | 0.845 | +1.06 |
|  | Somali | 1.038 | 0.947 | 0.963 | +1.72 |
|  | Benishangul-Gumuz | 0.882 | 0.820 | 0.822 | +0.25 |
|  | SNNPR | 0.902 | 0.821 | 0.827 | +0.69 |
|  | Gambela | 0.867 | 0.790 | 0.793 | +0.38 |
|  | Harari | 0.914 | 0.855 | 0.879 | +2.82 |
|  | Addis Ababa | 0.818 | 0.793 | 0.802 | +1.12 |
|  | Dire Dawa | 0.792 | 0.755 | 0.779 | +3.09 |
| Education | No education | Ref. | Ref. | Ref. | -- |
|  | Primary | 0.938 | 0.949 | 0.960 | +1.07 |
|  | Secondary | 0.927 | 0.947 | 0.960 | +1.38 |
|  | Higher | 0.888 | 0.904 | 0.907 | +0.27 |
| Wealth index | Poorest | Ref. | Ref. | Ref. | -- |
|  | Poorer | 0.950 | 0.882 | 0.897 | +1.62 |
|  | Middle | 0.853 | 0.781 | 0.799 | +2.24 |
|  | Richer | 0.756 | 0.725 | 0.749 | +3.29 |
|  | Richest | 0.675 | 0.665 | 0.684 | +2.86 |
| Religion | Orthodox | Ref. | Ref. | Ref. | -- |
|  | Catholic | 0.622 | 0.893 | 0.893 | −0.01 |
|  | Protestant | 1.054 | 1.181 | 1.163 | −1.53 |
|  | Muslim | 1.247 | 1.361 | 1.338 | −1.69 |
|  | Traditional | 1.451 | 1.522 | 1.515 | −0.43 |
|  | Other | 1.009 | 1.263 | 1.201 | −4.90 |
| Marital status | Married | Ref. | Ref. | Ref. | -- |
|  | Living with partner | 0.378 | 0.408 | 0.414 | +1.53 |
|  | Widowed | 0.531 | 0.429 | 0.436 | +1.64 |
|  | Divorced | 0.538 | 0.523 | 0.524 | +0.32 |
|  | Separated/no longer living together | 0.702 | 0.567 | 0.556 | −1.98 |
| Current contraceptive method | No current method | Ref. | Ref. | Ref. | -- |
|  | Modern method | 1.069 | 1.100 | 1.073 | −2.46 |
|  | Traditional/folkloric | 0.820 | 1.085 | 1.077 | −0.72 |

**Panel B: Model-fit and dispersion diagnostics**

| **Statistic** | **Conventional Poisson** | **Generalized Poisson** |
| --- | --- | --- |
| Analytical sample, n | 6,585 | 6,585 |
| Log likelihood | −7,068.356 | **−6,783.557** |
| No. estimated parameters | 36 | 37 |
| AIC | 14,208.71 | **13,641.11** |
| BIC | 14,453.24 | **13,892.44** |
| Generalized-Poisson dispersion parameter, α | -- | **−0.182** |
| Maximum absolute relative change in IRR* | -- | **4.90%** |

AIRR. Adjusted rate ratio; IRR, Incidence rate ratio; AIC, Akaike information criterion; BIC, Bayesian information criterion EMDHS, Ethiopian Mini Demographic and Health Survey.

Note. Reference categories were age 15–19 years, urban residence, Tigray region, no education, poorest wealth quintile, Orthodox religion, married, and no current contraceptive method. The Survey -adjusted poisson model was the primary inferential model and generalized Poisson Models used the same analytical sample and covariate specification and were fitted as likelihood-based sensitivity models to assess distributional assumptions. The generalized Poisson dispersion parameter was negative (α = −0.182), consistent with the under dispersion. Relative change was calculated between the conventional Poisson and generalized Poisson IRRs as [(IRR_GP – Irruption)/Irruption] × 100. Absolute relative changes were below 5% for all non-reference predictor categories, indicating that accommodation of underdispersion did not materially alter coefficient magnitudes. Likelihood-based fit statistics in Panel B are therefore compared only between conventional and generalized Poisson models and should not be interpreted as direct fit comparisons with the survey-adjusted primary model.
